# The potential role of aberrant microglial synaptic pruning in the neurodevelopmental pathogenesis of tourette syndrome

**DOI:** 10.3389/fnins.2026.1895330

**Published:** 2026-07-08

**Authors:** Lu Lu, Yuxuan Zang, Caiwei Song, Yan Wang

**Affiliations:** 1 Department of Pediatrics, The First Affiliated Hospital of Heilongjiang University of Chinese Medicine, Harbin, China; 2 Graduate School, Heilongjiang University of Chinese Medicine, Harbin, China

**Keywords:** basal ganglia circuitry, microglia, nanomedicine, neuroimmune microenvironment, synaptic pruning, tourette syndrome

## Abstract

Tourette syndrome is a neurodevelopmental disorder traditionally attributed to dopamine system hyperactivity within the cortico-striato-thalamo-cortical circuitry. However, classical neurotransmitter hypotheses fail to fully explain the spatiotemporal and developmental specificities of the disorder. Consequently, research focus has shifted toward the neuroimmune microenvironment, specifically the role of microglia. This review aims to comprehensively explore the potential mechanisms of microglia-mediated aberrant synaptic pruning in the pathogenesis of tourette syndrome and to evaluate emerging therapeutic strategies. Methodologically, the study employs a narrative review approach to synthesize current neuroimmunobiology literature to reconstruct the pathological trajectory from early immune dysregulation to targeted interneuron impairment. Additionally, it conceptually explores natural product active monomers through a multi-target network pharmacology framework and assesses the translational potential of engineered nanodelivery systems. The findings indicate that genetic susceptibilities, such as histidine decarboxylase gene mutations, interact with environmental stressors, like maternal immune activation, to induce a chronically primed state in basal ganglia microglia. These primed innate immune cells are hypothesized to execute excessive synaptic pruning against highly vulnerable parvalbumin-expressing fast-spiking interneurons, a process significantly facilitated by the pathological downregulation of presynaptic protective signals. The physical loss of this local gamma-aminobutyric acid-ergic inhibitory network attenuates feedforward inhibition on medium spiny neurons, potentially contributing to macroscopic dopaminergic disinhibition. To address these mechanisms, multi-target natural therapies delivered via intelligent nanoplatforms present a theoretically promising approach to penetrate the blood–brain barrier and reverse pathological microglial phenotypes. Ultimately, this manuscript proposes a perspective of tourette syndrome as a microstructural developmental disorder of the circuitry rather than a mere neurotransmitter imbalance, providing a critical theoretical foundation for developing precise, next-generation neuroimmune-modulating interventions.

## 1 Introduction

Tourette Syndrome (TS) is a neuropsychiatric disorder characterized primarily by multiple motor and vocal tics, and is highly comorbid with obsessive-compulsive disorder (OCD) and attention-deficit/hyperactivity disorder (ADHD) (Cavanna, 2022). For a long time, the hyperactivity of the dopamine system within the cortico-striato-thalamo-cortical (CSTC) circuit has been considered the primary cause driving the onset of the disease (Frey and Malaty, 2022). However, with the accumulation of large-sample clinical cohort data and the evolution of basic neurodevelopmental biology, this classic hypothesis is facing increasingly prominent bottlenecks in clinical and mechanistic exploration (Paschou et al., 2022; Johnson et al., 2023). Clinically, although traditional pharmacological treatments represented by D2 receptor antagonists can partially alleviate motor phenotypes, they are frequently limited by severe extrapyramidal reactions and metabolic disorders (Leucht et al., 2024). More critically, conventional monoamine-targeted therapies often fail to achieve optimal efficacy in a substantial proportion of treatment-resistant patients, highlighting the clinical complexity of these refractory cases (Müller-Vahl et al., 2024; Zhao et al., 2025d). Pathologically, a simple neurotransmitter imbalance is insufficient to fully explain the strict chronobiological characteristics of the disease (Koirala et al., 2024). Tic symptoms typically emerge during the preschool to early childhood period and show a tendency for spontaneous remission after the cerebral cortex matures in adolescence (Lowe et al., 2019). This highly age-dependent feature strongly suggests that the etiology of the disease is not a primary or transient abnormality in transmitter secretion, but rather stems from deep-seated changes in the microarchitecture of the central neural network during a critical period of brain development (Lamanna et al., 2023; Zouki et al., 2023). The prevailing pharmacological paradigm, which primarily relies on single-target interventions directed at downstream symptomatic expression, urgently requires a theoretical breakthrough to address the complex network pathophysiology of the disorder (Andrén et al., 2022).

Given the limitations of traditional neurochemical models in explaining the age-dependent onset and remission trajectories, recent research paradigms have increasingly shifted toward the neuroimmune microenvironment (Zhongling et al., 2022; Mahjoub and Martino, 2023). During the normal postnatal brain development process, microglia, the resident innate immune cells within the brain parenchyma, do not merely play a single role as pathogen scavengers (Paolicelli et al., 2022). They eliminate redundant synapses in early development through a precise synaptic pruning mechanism, thereby consolidating mature neural circuits (Soteros and Sia, 2022; Huo et al., 2024; Nandi et al., 2025). This physiological process relies heavily on the precise recognition of the classical complement cascade (e.g., the C1q-C3-CR3 pathway) on the presynaptic membrane by microglia, as well as the precise balancing of “do not eat me” signals (e.g., CD47) (Soteros and Sia, 2022; Huo et al., 2024). However, evidence from high-throughput sequencing and single-cell RNA sequencing (scRNA-seq) suggests that under the perturbation of specific environmental and genetic stress, this highly regulated microscopic process is highly susceptible to pathological deviation (Pettas et al., 2022; Fan et al., 2023a; Tillmon et al., 2024). Phagocytosis-related genes (e.g., Trem2, Apoe) within microglia exhibit abnormal upregulation, while homeostasis-maintaining genes (e.g., Cx3cr1, P2ry12) are significantly downregulated (Woodburn et al., 2021; Pettas et al., 2022). This deep phenotypic remodeling at the transcriptomic level may lead microglia to excessively prune synapses, thereby irreversibly altering the physical connection structure of normal neural circuits (Faust et al., 2021).

Under the specific pathological state of TS, abnormal synaptic pruning mediated by microglia is proposed as a critical conceptual link, potentially bridging upstream pathogenic factors and downstream neurotransmitter dysregulation. Recent studies on pedigrees with histidine decarboxylase (HDC) gene mutations provide an entry point with significant translational value: histamine is not only a conventional neurotransmitter but also a key immune brake molecule for microglia (Johnson et al., 2023). The loss of histamine signaling caused by HDC mutations may leave microglia in the basal ganglia in a chronic, low-threshold primed state (Jindachomthong et al., 2022). Upon this fragile microecological foundation, environmental factors such as maternal immune activation (MIA) or early peripheral infection may introduce peripheral pro-inflammatory cytokines into the central nervous system by disrupting the immature blood–brain barrier (BBB) (Ostrem et al., 2024; Tang et al., 2025).

Crucially, this neuroimmune microenvironment imbalance may possess high target specificity within the CSTC circuit (De Biase et al., 2017). A population of parvalbumin-positive (PV+) fast-spiking interneurons (FSIs) exists within the basal ganglia, which have extremely high metabolic demands and whose developmental maturation timing coincides precisely with the peak of childhood tic onset (Burton et al., 2024; Wang et al., 2024a). Under the continuous erosion of the abnormal immune microenvironment, the protective extracellular matrix (perineuronal nets, PNNs) surrounding these interneurons is highly susceptible to enzymatic degradation (Fawcett et al., 2019; Liu et al., 2024). This leads to the exposure of their inhibitory synapses, making them preferential phagocytic targets for the microglial complement system. Based on this, we propose that abnormal synaptic pruning by microglia does not overthrow the classic dopamine hypothesis, but rather provides a forward-looking explanatory basis for it: because microglia excessively eliminate the neural connection networks that locally release gamma-aminobutyric acid (GABA) for inhibitory signal transmission, the feedforward inhibition effect on medium spiny neurons (MSNs) in the direct pathway is weakened, ultimately leading to the macroscopic phenotype of disinhibition and excessive release in the downstream terminal dopamine system.

Based on the aforementioned theoretical evolution in neuroimmunobiology, this review aims to comprehensively explore the potential mechanism of abnormal microglial synaptic pruning in the pathogenesis of TS. This article outlines the pathological progression from early immune microenvironment imbalance and multicellular network cascade activation to the targeted impairment of specific interneurons.

## 2 Literature search strategy

To provide a comprehensive and unbiased overview of the neuroimmunological mechanisms in TS, a comprehensive literature search was conducted across PubMed, Web of Science, and CNKI databases from inception to May 2026. The search strategy incorporated combinations of the following core medical subject headings (MeSH) and keywords: “tourette syndrome” OR “tic disorders,” “microglia,” “synaptic pruning,” “complement cascade” (e.g., C1q, C3, CR3), “parvalbumin-positive interneurons,” “basal ganglia circuitry,” and “nanomedicine” OR “natural products.”

Literature inclusion focused on: (1) direct clinical and post-mortem evidence of neuroinflammation and interneuron loss in TS; (2) mechanistic studies utilizing TS-relevant animal models (e.g., Hdc knockout mice) or cellular systems; and (3) broader mammalian neurodevelopmental studies defining the fundamental pathways of microglia-mediated synaptic elimination. Generally, articles lacking clear relevance to the cortico-striato-thalamo-cortical circuitry were excluded; however, selected preprints and non-peer-reviewed repository items were exceptionally included when directly relevant to the core hypothesis and are clearly identified as such. This narrative review synthesizes these multi-layered findings to construct a novel hypothesis-generating framework.

## 3 Pathogenic triggers and microglial immune priming

During normal synaptic development, microglial pruning activity is subject to extremely strict spatiotemporal constraints by the central microenvironmental network (Scott-Hewitt et al., 2020). However, in the early pathological process of TS, microglia in the basal ganglia often deviate from immune homeostasis during critical developmental periods, entering a pre-existing abnormal condition known as the immune primed state (Wang et al., 2024a). Microglia in the primed state have undergone covert reprogramming at the transcriptomic level (Voshart et al., 2024). The activation thresholds of their pro-inflammatory receptors and phagocytosis-related molecules are significantly lowered, resulting in extreme sensitivity to subsequent changes in the microenvironment (Kwon et al., 2022). This decisive pathological transition is not spontaneous but is caused by the combined effect of the host’s genetic susceptibility defects and peripheral environmental stimuli during early development (Jiang et al., 2022). These two factors interact to disrupt local immune tolerance in the striatum, establishing the pathological foundation for the subsequent excessive synaptic pruning of specific interneurons (Frick, 2025).

### 3.1 Genetic susceptibility and the release of microglial immune brakes

TS possesses highly complex genetic heterogeneity. Recent genetic studies on HDC gene mutation pedigrees provide direct human clinical evidence for elucidating the underlying neuroimmune mechanisms of the disease (Jindachomthong et al., 2022). Furthermore, basic research utilizing TS-specific animal models, such as the Hdc knockout mouse, experimentally corroborates how histaminergic signal deprivation leads to microglial priming (Dürholz et al., 2025). Although a single HDC mutation represents a rare variant within the TS population, its primary scientific value lies in serving as a prototypical pathological model that explicitly elucidates the causal chain between histaminergic signal deprivation and microglial priming. At the population level, TS involves the complex interaction of multiple loci such as SLITRK1 and CNTNAP2 (Domènech et al., 2021). Although these genetic defects possess diverse initial biological functions, their pathological effects exhibit significant functional convergence within the striatal microenvironment (Fichna et al., 2024). Specifically, by disrupting neuron–glia molecular communication, local metabolic homeostasis, or synaptic adhesion networks, these polygenic variations collectively lead to the downregulation of the activation threshold for basal ganglia microglia (Pankratz et al., 2024). The existence of this convergent pathological pathway suggests that the impaired immune defense mechanism derived from the HDC model might transcend certain single-gene defects (Jain et al., 2026). Therefore, the HDC mutation model functions as a proof-of-concept mechanistic prototype rather than a universal genetic blueprint. However, considering that HDC mutations are rare and do not account for the majority of TS cases, histaminergic deficiency and microglial disinhibition should not be interpreted as universal pathogenic mechanisms for all patients. Instead, this model vividly illustrates one specific trajectory of how diverse polygenic hits could potentially converge upon microglial dysregulation within a highly heterogeneous disease spectrum.

#### 3.1.1 HDC mutation and remodeling of the central histamine metabolic pathway

Under physiological conditions, the cell bodies of central histaminergic neurons are primarily clustered in the tuberomammillary nucleus (TMN) of the hypothalamus, with their nerve fibers projecting extensively to the basal ganglia (Han et al., 2025). Within this region, histamine is not only a monoamine neurotransmitter regulating arousal and cognition but also a key immune brake molecule for microglia (Erickson et al., 2020). HDC mutations directly lead to severe impairment of central histamine synthesis (Li et al., 2023). This congenital defect in the metabolic enzyme completely disrupts the local histamine concentration gradient in the striatum (Koski et al., 2020). The steady-state high-concentration histamine pool originally maintained in the basal ganglia is depleted, resulting in a physical regression of the immunosuppressive barrier in the microenvironment (Zhu et al., 2025). The continuous absence of histamine signaling essentially eliminates the physiological immune constraints on basal ganglia microglia (Carthy and Ellender, 2021). Deprived of the buffering effect of this brake molecule, the resident innate immune cells exhibit extremely high sensitivity to minor stimuli, and the stress resilience of the regional microenvironment is significantly reduced (He et al., 2024).

#### 3.1.2 Histamine receptor-mediated impairment of inward signal transduction in microglia

Histamine’s exertion of its immune brake function is highly dependent on the specific histamine receptor network on the microglial surface (Zhou et al., 2024). This primarily involves H1R, H2R, and H3R within the G protein-coupled receptors (GPCRs) family (Panula, 2021). At the physiological baseline level, the binding of histamine to specific receptors (such as H2R coupled to Gs proteins) continuously activates adenylate cyclase (Hatipoglu et al., 2023). This process maintains the dynamic balance of the cyclic adenosine monophosphate (cAMP)-protein kinase A (PKA) pathway within microglia, thereby exerting a tonic inhibition on pro-inflammatory factors such as nuclear factor kappa B (NF-κB) at the transcriptional level. When HDC mutations lead to persistent ligand deprivation, the signaling network downstream of the receptors collapses abruptly (Verweij et al., 2020). The abnormal fluctuation of cAMP concentration directly relieves the basal inhibition of pro-inflammatory transcription factors (Yarwood, 2020). Subsequently, significant transcriptomic reprogramming occurs within the nucleus (Höring et al., 2021). Microglia deviate from homeostasis, and the transcriptional thresholds for phagocytosis-related receptors, such as complement receptor 3 (CR3), and inflammatory chemokines are drastically lowered (Huo et al., 2024). Ultimately, microglia deprived of targeted constraints remain in a chronic, low-activation-threshold primed state (Lima et al., 2022). Although this genetic susceptibility alone is insufficient to directly trigger large-scale neuronal damage, it establishes the pathological foundation for the subsequent excessive synaptic pruning of specific interneurons (Borreca et al., 2024).

### 3.2 Environmental stress-induced spatiotemporal vulnerability and BBB disruption

While genetic susceptibility establishes the foundation for priming, environmental immune stress during early development constitutes the key exogenous factor inducing abnormal microglial pruning (Fernández de Cossío et al., 2021). Extensive epidemiological and clinical observations have confirmed that MIA and specific bacterial infections during early infancy are closely associated with the outbreak or abrupt exacerbation of tic symptoms (Wu et al., 2025c). However, it must be emphasized that the detailed molecular mechanisms discussed below regarding how these environmental factors disrupt the neurovascular unit and prime microglia are primarily mechanistic extrapolations. These processes are derived from broader neurodevelopmental models (such as ASD) and tic-related conditions (such as PANDAS/PANS), rather than direct human TS tissues. During the critical period of neurodevelopment from the embryonic stage to the early postnatal stage, the neurovascular unit (NVU) is not yet fully mature, and the BBB possesses physiological high permeability and spatial vulnerability (Smith et al., 2022). Furthermore, it is essential to recognize that the evidence linking maternal immune activation, early infections, and PANDAS/PANS-related mechanisms to TS remains complex and highly controversial (Mahjoub and Martino, 2023). The clinical and epidemiological data are heterogeneous, and alternative interpretations persist regarding whether these events trigger an active, continuous neuroimmune cascade or merely act as non-specific risk modifiers that transiently unmask underlying circuit vulnerabilities (Han et al., 2021; Jiang et al., 2022). Consequently, the environmental triggers discussed herein should not be viewed as a unified or deterministic disease pathway for all TS cases, but rather as contextual stress factors that may drive disease pathogenesis in a specific subset of vulnerable individuals.

#### 3.2.1 Long-term epigenetic imprinting of fetal microglial phenotypes by MIA

MIA during pregnancy, induced by infection, is a significant cause of perturbation in the central neuroimmune homeostasis of offspring (Tang et al., 2025). During this process, abnormally elevated pro-inflammatory cytokines in maternal peripheral blood (such as IL-6 and IL-17a) can penetrate the placental barrier and further cross the underdeveloped fetal BBB, directly reaching the developing brain parenchyma (Tang et al., 2025). Exogenous inflammatory signals bind to receptors on primitive microglia, triggering the abnormal activation of core intracellular transcription factors such as signal transducer and activator of transcription 3 (STAT3) (Li et al., 2025b). More crucially, this early inflammatory stimulus not only induces short-term cellular release effects but also leaves long-term epigenetic imprints in microglia (Lan et al., 2026). Inflammatory signal-mediated intracellular cascades specifically alter histone modification states (e.g., increased H3K4me3) or DNA methylation patterns in the promoter regions of phagocytosis- and inflammation-related genes (such as chemokine receptors and complement molecules) (Hamagami et al., 2026). This significant transcriptomic rearrangement shapes the innate immune memory of microglia (Wu et al., 2025d). Consequently, microglia persist in a covert primed state long after MIA resolution, pre-establishing a pathological predisposition for aberrant synaptic pruning in childhood (Loewen et al., 2023).

#### 3.2.2 Early peripheral infection and spatiotemporal coupling impairment of NVU permeability

Beyond embryonic MIA exposure, peripheral pathogen infection during early infancy constitutes a key second hit driving the comprehensive activation of microglia (Han et al., 2021). At this developmental stage, the integrity of the BBB relies heavily on the tight apposition among endothelial cells, pericytes, and astrocyte endfeet within the NVU (Naranjo et al., 2022). The systemic inflammatory storm induced by severe peripheral infection can act directly on the luminal receptors of brain microvascular endothelial cells, rapidly upregulating the expression and activity of matrix metalloproteinases (especially MMP-9) in endothelial cells and pericytes (Xin et al., 2025). Highly active MMP-9 not only degrades the core components of the vascular basement membrane but also precisely recognizes and hydrolyzes key tight junction proteins (such as Claudin-5 and ZO-1) that maintain the sealing of endothelial cell gaps (Vittal Rao et al., 2021). Under this enzymatic degradation stress, the structural support function of pericytes is severely impaired, leading to a substantial collapse of the physical barrier function (Qiu et al., 2021).

**Figure 1:**
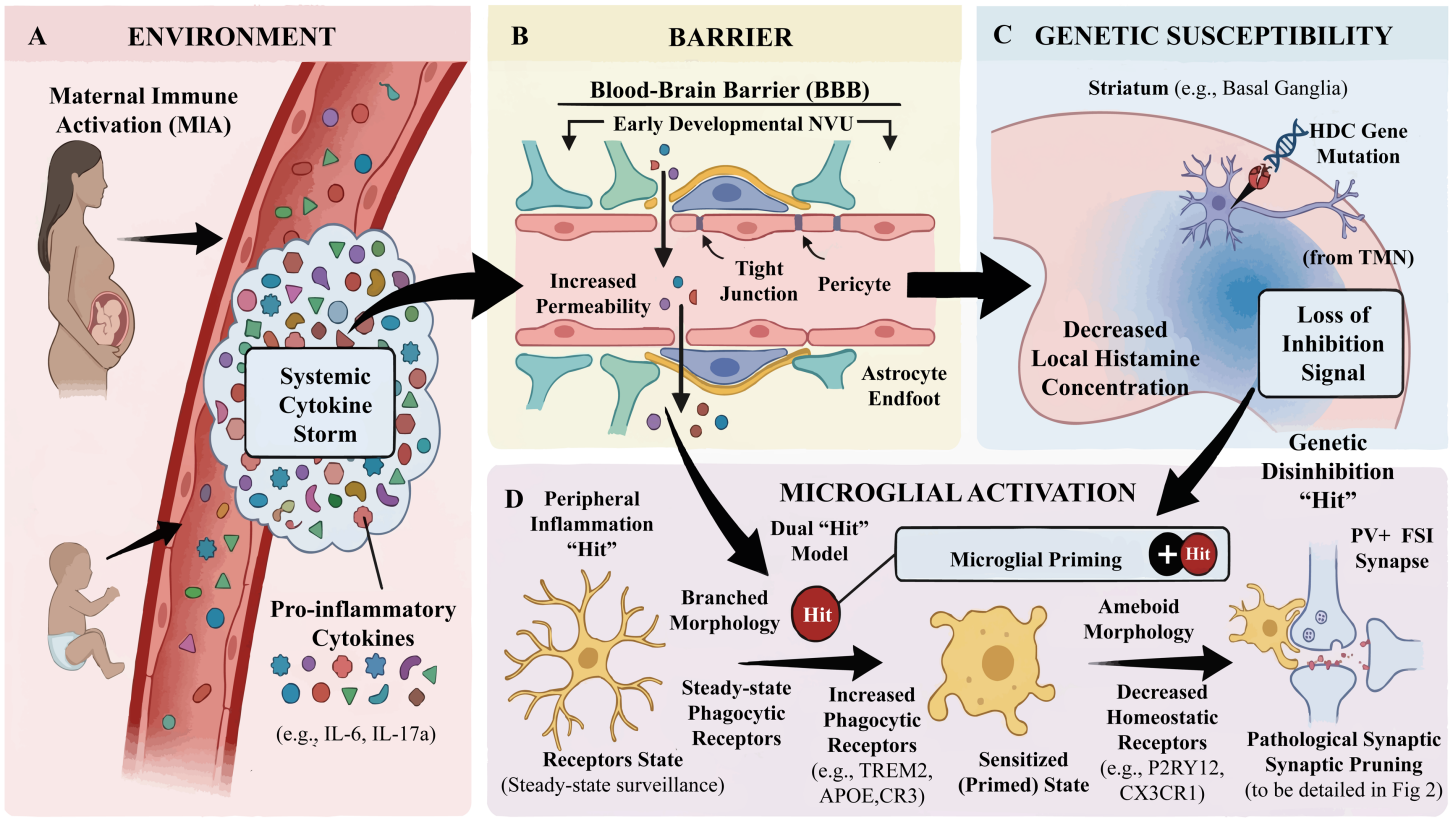
The proposed pathological cascade of microglial priming and aberrant synaptic pruning in TS. (A) Environmental immune stress. MIA during pregnancy or early postnatal infections induce a systemic cytokine storm, leading to elevated levels of peripheral pro-inflammatory cytokines (e.g., IL-6, IL-17a) (Iitani et al., 2024). **(B)** Neurovascular vulnerability and barrier dysfunction. The immature BBB within the early developmental NVU exhibits physiological permeability (Gong et al., 2025). Systemic inflammation further degrades tight junctions, facilitating the transcytosis and infiltration of peripheral cytokines into the striatal parenchyma (Gryka-Marton et al., 2025). **(C)** Genetic susceptibility and immune brake failure. HDC gene mutations impair histamine synthesis, drastically depleting the local histamine pool in the basal ganglia and depriving microglia of their constitutive inhibitory signals (Jindachomthong et al., 2022). **(D)** Cascade activation of microglia. The convergence of genetic disinhibition and exogenous inflammatory infiltration forces microglia to transition from a resting, branched morphology into a hyperactive, ameboid state (Mastenbroek et al., 2024). This phenotypic remodeling is characterized by the robust upregulation of phagocytic receptors (e.g., TREM2, APOE, CR3) and the downregulation of homeostatic markers (e.g., P2RY12, CX3CR1), ultimately precipitating targeted pathological pruning of PV + FSI synapses (Serrano-Pozo et al., 2021).

When the body encounters the aforementioned severe peripheral infection, massive pro-inflammatory cytokines released by the immune system can easily cross the damaged, incomplete BBB and directly invade the striatal parenchyma (Müller and Di Benedetto, 2025). Upon entering the central nervous system, these peripheral inflammatory signals rapidly initiate a cascade reaction with microglia already in a primed state due to genetic mutations (Zhao et al., 2025a). The release of genetic immune constraints and the amplification of peripheral inflammatory signals form a double hit, driving a significant phenotypic transformation in microglia (Figure 1). Microglia no longer maintain a ramified resting state; their morphology and function undergo fundamental changes, massively producing phagocytosis-related receptors and transitioning into a hyperactive pruning mode, thereby initiating the pathological destruction of neural circuits in specific brain regions (Borreca et al., 2024).

### 3.3 Horizontal pathological differentiation of neurodevelopmental disorders

Abnormal synaptic pruning mediated by microglia is not unique to TS; it also plays a key role in highly comorbid childhood neurodevelopmental abnormalities such as OCD and autism spectrum disorder (ASD) (Hao et al., 2024). To clarify the specificity of this immunopathological mechanism within different disease networks, it is necessary to horizontally differentiate TS from other developmental disorders with overlapping developmental stages and high clinical comorbidity (McHugh et al., 2026). Although the intrinsic cause of these diseases is neuroimmune system imbalance, and they all exhibit similar priming responses upon exposure to external environmental stimuli, there are highly subtle differences in the specific cell types they attack and the affected brain region circuits (Anderson et al., 2026).

Under the dual drive of genetic susceptibility and environmental triggers, the synaptic pruning targets of different diseases exhibit significant brain region heterogeneity (Faust et al., 2021). The microenvironmental imbalance of ASD corresponds to the cerebral cortex and hippocampal regions; its abnormal microglial activity tends to involve excitatory pyramidal neurons, and the core pathological time window usually opens in the very early postnatal period, ultimately leading to complex social interaction deficits and restrictive behaviors (Matuleviciute et al., 2023). Although OCD and TS both belong to the impairment category of the CSTC circuit and are both subject to similar exogenous influences such as streptococcal infection, their microscopic targets are entirely different (Younger, 2025). The immune remodeling of OCD is concentrated more in the cognitive-affective microcircuits of the orbitofrontal cortex and caudate nucleus, and it is speculated that the impaired nodes are mostly the synapses of MSNs mediating behavioral switching (Singh et al., 2023).

In contrast, the neuroimmune pathology of TS is highly localized to the motor control center of the basal ganglia (Liberati and Perrotta, 2024). Under specific backgrounds such as HDC mutations, FSIs with high metabolic features within the striatum may become the specific targets most vulnerable to microglia (Jindachomthong et al., 2022). TS can be distinguished from other comorbid disorders, forming characteristic motor and vocal tic symptoms, due to the extreme precision in its lesion location, timing of onset, and impaired neuron types (Wang et al., 2024a). In-depth analysis of the multiple differences among these developmental disorders in the dimensions of the microenvironment and specific circuits further highlights the core status of basal ganglia-specific abnormal pruning in the pathogenesis of TS (Table 1) (Liberati and Perrotta, 2024).

**Table 1:** Comparison of proposed microenvironmental and circuitry pathological characteristics among neurodevelopmental disorders.

Pathological and clinical dimensions	TS	OCD	ASD
Representative genetic susceptibility variants	Highly polygenic (e.g., rare HDC gene mutations)	Highly polygenic (e.g., SAPAP3, SLITRK1 variants)	Highly polygenic (e.g., SHANK3, MECP2 variants)
Core immune and environmental stressors	MIA, early streptococcal infections (PANDAS/PANS)	Early streptococcal infections (PANDAS), severe psychological stress	MIA, prenatal exposure to environmental toxins
Implicated characteristically affected brain regions	Basal ganglia motor circuitry (predominantly the putamen)	Orbitofrontal cortex-caudate nucleus cognitive circuitry	Widespread cerebral cortical regions, hippocampus
Hypothesized impaired neuronal subtypes	Local GABAergic FSIs	MSNs within the caudate nucleus or specific cortical projection neurons	Predominantly cortical excitatory pyramidal neurons
Proposed core period of aberrant pruning	Preschool to childhood	Childhood to early adolescence	Early postnatal to infancy
Primary core clinical phenotypes	Multiple involuntary motor and vocal tics	Recurrent intrusive obsessions and compulsions	Social communication deficits and early stereotypical behaviors

It is critical to emphasize that TS, OCD, and ASD are profoundly heterogeneous clinical spectrums. The genetic variants, implicated circuits, and neuronal subtypes listed here represent proposed prototypical mechanisms utilized to conceptually differentiate these overlapping disorders, rather than universal or rigid diagnostic blueprints for all patients. Adapted from Hall et al. (2025) and Hall et al. (2025).

## 4 Molecular execution of synaptic pruning and circuit remodeling

Positing microglial immune priming as a critical early trigger, the pathological process is hypothesized to enter a phase of substantial microscopic destruction (Bano et al., 2024). It is crucial to note that while the behavioral and macroscopic circuit-level disinhibition in TS is well-documented, the precise microscopic execution of complement-dependent synaptic pruning detailed below represents an inferred mechanistic framework. The sequence of molecular events, such as complement deposition and the downregulation of inhibitory signaling molecules, is primarily drawn from general microglial biology, neurodegenerative models, and stress-induced plasticity paradigms (Faust et al., 2021; Huo et al., 2024). These mechanisms await direct validation in TS-specific post-mortem human tissue or validated TS cellular systems. Whether innate immune cells in the primed state can eliminate target synapses hinges on whether the precise local molecular recognition mechanisms are disrupted (Gan et al., 2023).

### 4.1 Abnormal molecular recognition mechanisms lead to homeostatic imbalance in the microenvironmental ecosystem

When microglia are in a highly primed state, their phagocytic behavior is controlled by the competitive mechanism between the complement cascade and anti-phagocytic signals, while the pro-inflammatory microenvironment of the striatum leads to comprehensive disruption of the cell surface molecular marker system (Hu and Gao, 2025).

#### 4.1.1 Local abnormal enrichment of the classical complement cascade (C1q-C3-CR3) and amplification of phagocytic signals

Abnormal activation of the classical complement pathway is the core execution pathway of pathological synapse elimination (Xu et al., 2025). Stimulated by microenvironmental inflammatory factors, the C1q protein, serving as the initiating recognition molecule, specifically anchors to the stressed presynaptic membrane (Zhao et al., 2025b). The local enrichment of C1q rapidly recruits and activates C3 convertase. This enzyme locally cleaves massive amounts of free complement C3 into active fragments (such as C3b and iC3b) at the synapse (Zhao et al., 2025c). These fragments densely deposit on the synaptic surface, forming potent pathogenic “eat me” signals. The highly expressed CR3 on the microglial surface specifically recognizes these deposited fragments (Cai et al., 2026b). Receptor-ligand binding directly initiates cytoskeletal rearrangement, driving the formation of the phagocytic cup and the endocytic degradation process (Han et al., 2024).

#### 4.1.2 Defensive stripping of the presynaptic “do not eat me” signal (CD47-SIRPα)

While the complement system is abnormally amplified, the intrinsic molecular defense barrier of the synapse undergoes significant collapse. Under physiological conditions, the CD47 molecule expressed on the presynaptic membrane transmits inhibitory signals by binding to the SIRPα receptor on the microglial surface, effectively preventing non-specific phagocytosis (Shui et al., 2022). Under highly primed immune stress, we hypothesize that the expression level of CD47 molecules on the surface of target synapses exhibits significant downregulation (DeVries et al., 2024). While direct post-mortem evidence in TS is currently lacking, this process is speculated to be controlled by transcriptional repression caused by stress-induced NF-κB pathway activation, or ectodomain shedding mediated by local highly active metalloproteinases (Wu et al., 2025c). The loss of CD47 renders the normal immune evasion mechanism completely ineffective, leaving the synapse in a state of defenseless exposure (Han et al., 2024).

#### 4.1.3 Chemotactic crosstalk between astrocytes and microglia (the ATP-P2RY12 axis)

**Figure 2:**
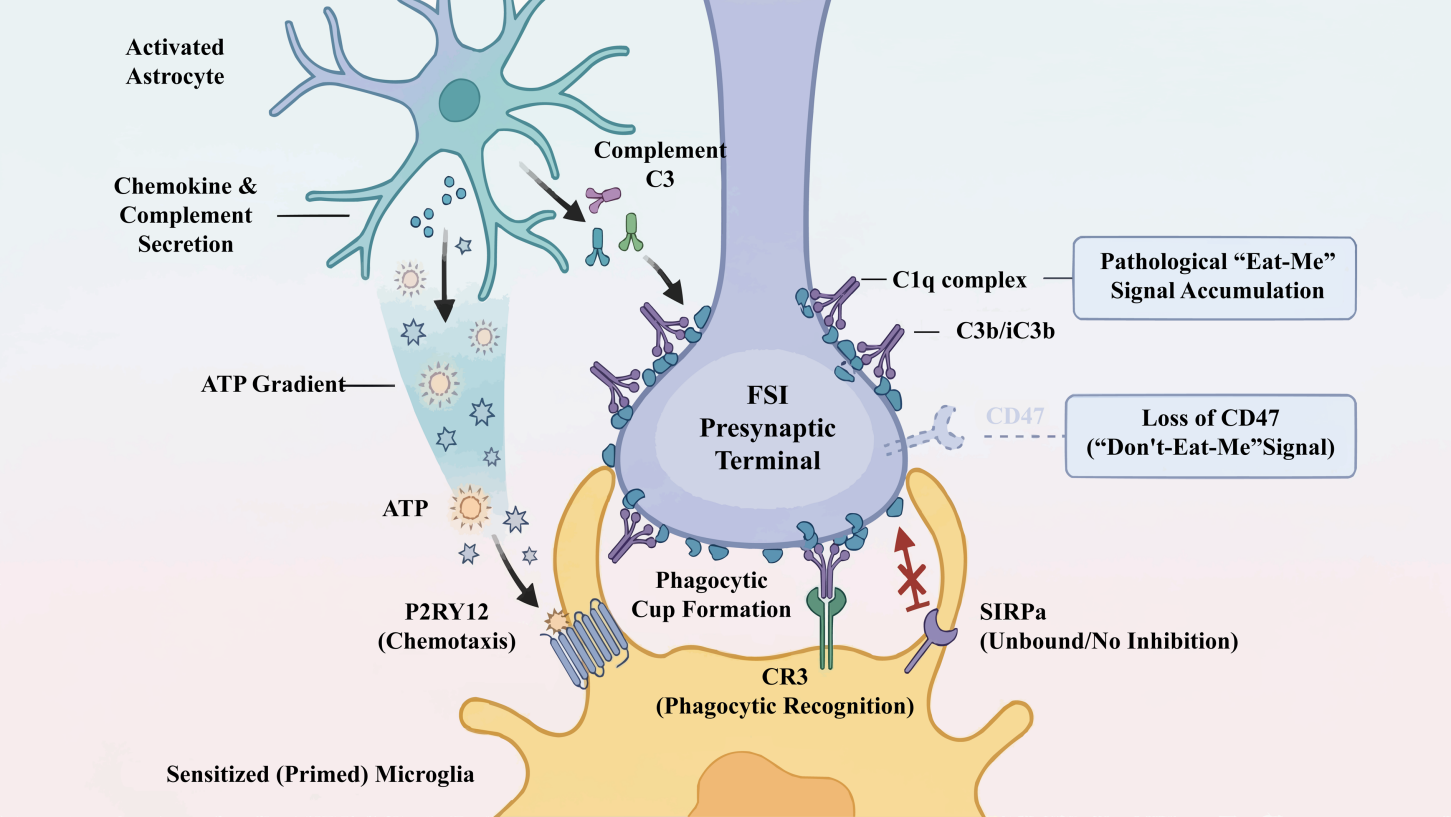
Hypothesized multicellular cascade and molecular execution mechanisms of aberrant synaptic pruning. Activated astrocytes secrete ATP and complement C3 into the local microenvironment. The ATP gradient establishes a chemotactic signal, guiding sensitized microglia towards the target synapse via P2RY12 receptors. Concurrently, pathological “eat-me” signals accumulate on the FSI presynaptic terminal, characterized by the dense deposition of the C1q complex and active C3 fragments (C3b/iC3b). Furthermore, the expression of CD47 on the FSI presynaptic membrane is pathologically downregulated. The absence of this “do not-eat-me” signal leaves the microglial SIRPα receptor unbound, neutralizing the physiological inhibitory signaling. Directed by the ATP-P2RY12 chemotactic axis and the lack of SIRPα-mediated inhibition, sensitized microglia utilize CR3 to specifically recognize the deposited C3b/iC3b fragments. This receptor-ligand binding directly initiates phagocytic cup formation and the structural elimination of the FSI presynaptic terminal. [The mechanisms of ATP-P2RY12 chemotaxis, complement-dependent synaptic pruning, and CD47/SIRPα signaling depicted in this figure are derived from Cai et al. (2026a), Nandi et al. (2025), and Zhang et al. (2024). This sequence is extrapolated from general microglial biology and remains an inferred framework pending direct visualization in TS-specific synapses].

The progression from molecular recognition to circuit disruption follows a well-defined three-step cascade tri-phasic cascade within the striatal multicellular network. This pathological trajectory initiates with a priming phase, where early-life immune stressors (such as MIA) induce latent epigenetic reprogramming in microglia, lowering their activation threshold without triggering immediate phagocytosis (Cathomas et al., 2023). The process advances to a recruitment and tagging phase during subsequent peripheral inflammatory challenges (Lichtenstein et al., 2025). In this stage, reactive astrocytes—serving as the primary sensors of microenvironmental perturbation—precede microglial action by secreting high concentrations of ATP to establish a chemotactic gradient (Saito et al., 2024). Concurrently, these astrocytes augment the local pool of complement C3, which specifically deposits onto the stressed synapses of FSIs (Spencer et al., 2025). Finally, the sequence culminates in the execution phase, where the pre-sensitized microglia, guided by the AST-derived ATP gradient via P2RY12 receptors, trans-spatially migrate to the tagged synapses (Wu et al., 2025c). The interaction between microglial CR3 and synaptic C3/C1q, combined with the loss of CD47-mediated inhibitory signaling, switches the microenvironment from a homeostatic surveillance state to a pathological stripping mode (Han et al., 2024). This refined temporal logic transforms the previously ambiguous cellular crosstalk into a structured, hypothetical sequential pathway (Figure 2).

### 4.2 Extremely high metabolic load and loss of physical shielding induce the pathological vulnerability of interneurons

The abnormal activation of synaptic pruning exhibits extremely high spatial specificity within the striatal microenvironment, a phenomenon driven by the convergence of localized neurovascular vulnerability and region-specific extracellular matrix properties (Guo and Kumar, 2026). While PV + FSIs are widely distributed throughout the brain, their preferential impairment in the basal ganglia is governed by a distinct pathological ‘double-lock’ mechanism. During the critical childhood window of TS pathogenesis, the NVU of the striatum maintains a state of physiological immaturity and higher permeability relative to the cerebral cortex, thereby creating a localized entry point for peripheral inflammatory stressors and cytokines (Neurath, 2024). Furthermore, the PNNs surrounding striatal FSIs possess a unique biochemical signature—characterized by a specific chondroitin sulfate proteoglycan (CSPG) profile and sulfation patterns—that renders them exceptionally susceptible to the enzymatic activity of MMP-9, which is markedly elevated in the TS-primed microenvironment (Wu et al., 2025c). This regional susceptibility, exacerbated by the extreme mitochondrial metabolic load and resulting oxidative stress inherent to the fast-spiking phenotype, ensures that the initial complement-mediated tagging (C1q/C3) is concentrated within the motor control circuits of the basal ganglia (Wong et al., 2026). The localized enzymatic collapse of these physical shields leaves striatal synapses structurally naked and defenseless, directly precipitating the targeted elimination of this inhibitory network by hyperactive microglia while sparing similar neuronal populations in other brain regions (Liu et al., 2025). It must be explicitly stated that direct *in vivo* evidence demonstrating microglial engulfment of PV + FSI synapses, specific complement deposition on these terminals, or a proven causal link between synaptic pruning and tic-like phenotypes in TS-specific models remains unestablished. Therefore, the preferential elimination of PV + FSIs discussed here should be regarded as a proposed conceptual model—extrapolated from their unique metabolic vulnerability and neuroinflammatory literature—rather than an experimentally confirmed pathogenic process in TS (Burton et al., 2024; Liu et al., 2025; Wu et al., 2025c). Importantly, while robust preclinical evidence from related neuroinflammatory and stress models demonstrates that pharmacological inhibition of matrix metalloproteinases (e.g., MMP-9) preserves PNN integrity and subsequently prevents PV + interneuron loss (Fawcett et al., 2019), whether this exact protective causality holds true in TS-specific in vivo models remains a critical hypothesis awaiting empirical validation.

### 4.3 Physical loss of local inhibitory networks drives the disinhibition effect of neural circuits

As microglia continuously and precisely phagocytose the synapses of specific interneurons, this structural destruction at the microscopic level ultimately exceeds the local self-repair capacity, directly leading to functional impairment of the basal ganglia core circuit at the macroscopic level (Pulvermüller et al., 2021).

#### 4.3.1 Attenuation of GABAergic feedforward inhibition and action potential threshold variation in MSNs

In the classic CSTC circuit, FSIs exert extremely potent GABAergic feedforward inhibition on the massive number of output nodes—MSNs—through dense local axon collaterals (Brady et al., 2022). Abnormal synaptic pruning leads to the structural physical loss of the inhibitory synapses of FSIs (Fan et al., 2023b; Nandi et al., 2025). The sharp decrease in GABA release in the synaptic cleft directly reduces the binding rate of GABA-A receptors on the postsynaptic membrane of MSNs (Wang et al., 2024b; Guo and Kumar, 2026). Chloride ion influx is significantly reduced, causing the resting membrane potential of MSNs to undergo abnormal depolarization (Day et al., 2024). The threshold for MSNs to generate action potentials is drastically lowered, leading to the complete collapse of the local excitation/inhibition (E/I) dynamic balance in the striatum (Burton et al., 2024).

#### 4.3.2 Trans-synaptic Cascade amplification of disinhibited abnormal dopamine release

**Figure 3:**
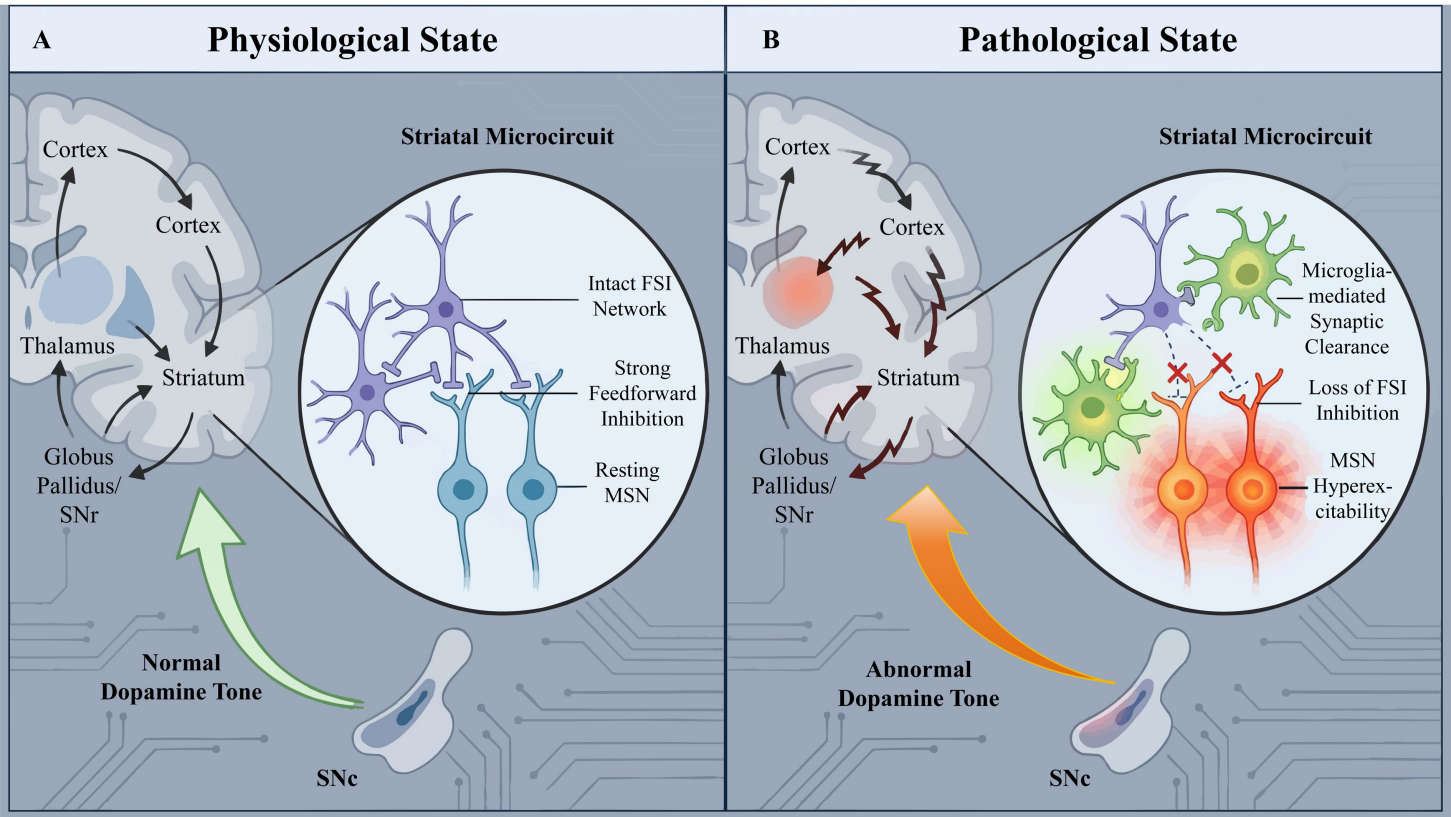
Hypothesized circuit-level disinhibition driven by microglia-mediated structural loss of FSI synapses. **(A)** Physiological State. The intact FSI network provides robust feedforward inhibition to MSNs, maintaining local striatal microcircuit homeostasis and a normal dopamine tone from the SNc. **(B)** Pathological state. Hyperactive microglia execute targeted synaptic clearance against FSIs, physically eliminating their inhibitory projections (Wang et al., 2023). The structural loss of FSI-mediated feedforward inhibition directly induces significant MSN hyperexcitability. This localized disruption of the excitation/inhibition balance triggers a trans-synaptic cascading disinhibition effect across the broader cortico-striato-thalamo-cortical circuitry, ultimately driving an abnormal, excessive dopamine release from the SNc and the macroscopic hyperactive signaling phenotype (Qian et al., 2022).

The hyperactivity of local MSNs within the striatum leads to a stepwise amplification of electrical signals along the circuit (Lin et al., 2021). Deprived of the constraints of feedforward inhibition, MSNs overactivate both the direct and indirect pathways, resulting in a loss of control over the output signals from the basal ganglia to the thalamus and cortex (Worbe and Pasquereau, 2026). Driven by this aberrant cortical feedback, the reverse compensatory enhancement of cortico-striatal excitatory projections strongly activates local cholinergic interneurons (CINs). This local microcircuit dynamic effectively bypasses the somatic constraints of the nigrostriatal dopaminergic system (Lemos et al., 2026). This cascade pathological drive induces an explosive, action-potential-independent release of terminal dopamine (Figure 3) (Wu et al., 2025b). At this point, the microstructural remodeling mechanism establishes an irreversible pathological foundation for the dopamine hyperfunction phenomenon (Xi et al., 2022).

#### 4.3.3 Clinical implications: reinterpreting stimulant-induced tic exacerbation in comorbid ADHD

The clinical utility of our proposed microstructural model is most vividly demonstrated when examining the intersection of TS with ADHD. Given the exceptionally high comorbidity rate (approximately 50–60%) between TS and ADHD (Koirala et al., 2024), a persistent clinical dilemma is that central stimulants, such as amphetamines, prescribed for ADHD frequently exacerbate tic symptoms. While the classic dopamine hypothesis attributes this to a general hyperdopaminergic state, it lacks microcircuit-level specificity.

Our microglial pruning model offers a novel, structural explanation for this specific pharmacological vulnerability. Under physiological conditions, the robust GABAergic feedforward inhibition provided by PV + FSIs acts as a dynamic gatekeeper, effectively buffering fluctuations in striatal dopamine. However, in the TS microenvironment, hyperactive microglia have physically eliminated these critical inhibitory synapses. Consequently, when amphetamines are administered, they act upon a dopamine system that has been structurally stripped of its normal GABAergic constraints. The exogenous dopaminergic stimulation further enhances an already dysregulated, disinhibited nigrostriatal pathway. Without the intrinsic FSI-mediated brake mechanism, dopamine signals cascade unbuffered through the basal ganglia, directly translating into amplified motor output and exacerbated tic severity (Worbe and Pasquereau, 2026). This crucial intersection elegantly harmonizes the neuroimmune microstructural damage hypothesis with the classic dopamine phenotype, illustrating how early-life inflammatory pruning establishes the precise vulnerability for later pharmacological hypersensitivity.

## 5 A new therapeutic paradigm targeting the neuroimmune microenvironment

Based on the hypothesized link between abnormal microglial synaptic pruning and neural circuit impairment in TS, therapeutic strategies targeting neuroimmune interactions represent a speculative translational extension aimed at reconstructing the central microenvironmental landscape and reversing the pathological pruning state (Ranjan et al., 2024). Its core concept is to utilize neuroimmune regulatory mechanisms to efficiently transform the impaired striatal microenvironment during critical developmental periods from a pathological excessive pruning mode to a physiological homeostatic protective mode (Wang et al., 2023). This transformation is not only expected to directly protect key inhibitory interneurons from physical destruction but also, by correcting immune stress signals at the source, creates opportunities for synergistic enhancement with traditional pharmacological interventions, achieving an upgrade from symptom control to disease-modifying therapies (McCrea et al., 2025). It must be clarified that the multi-target and smart nanodelivery strategies proposed in the following sections are conceptualized from broader neuroinflammatory and drug delivery fields. While theoretically promising, their specific efficacy and spatial targeting require rigorous validation in TS-relevant *in vivo* models. Conceptually, the ideal therapeutic window for such anti-pruning interventions strictly aligns with the early onset phase of tics—typically between ages 4 and 7 (Greydanus and Tullio, 2020). Intervening during this critical early-childhood period aims to halt aberrant microglial hyper-phagocytosis before the irreversible physical elimination of interneurons reaches its peak during pre-adolescence (Bloch and Leckman, 2009).

### 5.1 Traditional single-target immunosuppressive strategies face translational dilemmas in the complex brain microenvironment

Current pharmacological attempts targeting central neuroinflammation mostly focus on broad-spectrum immunomodulators such as minocycline; however, such interventions face enormous challenges in their translation to clinical application (Virani et al., 2023). Although some broad-spectrum drugs can alleviate short-term symptoms by blocking specific pro-inflammatory signaling pathways, they are speculated to be unable to achieve persistent microenvironmental remodeling in the highly complex brain microenvironment (Walgaard et al., 2011). Monotherapies strictly confined to a single molecular target tend to disrupt holistic neuroimmune homeostasis. Such interventions not only run the risk of triggering robust compensatory immune networks but may also inadvertently dismantle the intrinsic defensive barriers of the central nervous system through excessive functional inhibition (Paolicelli et al., 2022; Tansey et al., 2022). More critically, because the central nervous system is highly dynamic during the developmental window, non-specific immunosuppression is speculated to interfere with the normal synaptic pruning process, thereby facing the risk of side effects such as inducing neurodevelopmental delay or secondary cognitive impairment. This intervention modality, lacking targeting precision and network depth, fails to address the multicellular collaborative pathological pattern of TS (Mohammady et al., 2026). To overcome these pharmacological bottlenecks, an ideal disease-modifying therapeutic paradigm must satisfy three stringent translational criteria: (1) robust blood–brain barrier (BBB) penetrability to effectively reach striatal lesions; (2) multi-target synergistic capacity to simultaneously suppress microglial hyper-phagocytosis and restore presynaptic defensive signals (McCrea et al., 2025); (3) high spatiotemporal selectivity to avoid interfering with physiological synaptic pruning during critical developmental windows. Meeting these requirements necessitates a transition from single-node intervention to a systematic, network-based regulatory approach (Huo et al., 2024).

### 5.2 Multi-target network pharmacology exhibits significant potential in remodeling the neuroimmune microenvironment

Guided by the aforementioned criteria, active monomers from natural products based on the framework of network pharmacology emerge as a highly rational alternative (Sun et al., 2025). Distinct from the mechanical blockade of single-target synthetic drugs, these natural compounds possess an inherent pleiotropic corrective potential (Jiang et al., 2023). This enables them to simultaneously intervene in multiple core molecular nodes within the pathological signaling chain of TS, effectively shifting the striatal microenvironment from a pro-inflammatory state toward homeostatic protection (Sun et al., 2025).

#### 5.2.1 Natural monomer-mediated phenotypic reprogramming toward homeostasis

Multi-target compounds such as baicalin and gastrodin can directly intervene in the inward signal transduction network of microglia (Sun et al., 2025). At the molecular level, these monomers can specifically bind to or downregulate Toll-like receptors (TLRs) on the microglial surface, thereby potently blocking the nuclear translocation process of the downstream NF-κB signaling pathway (Yang et al., 2024a). The inhibition of pro-inflammatory transcription factors directly attenuates the transcriptional drive of amoeboid microglia for phagocytic receptors and chemokines (Phan Van et al., 2024). Concurrently, such natural monomers can prompt phenotypic state transition of microglia by activating antioxidant pathways such as Nrf2/HO-1 (He et al., 2021). Their morphology is substantially reversed from a highly invasive amoeboid state to a ramified homeostatic state with physiological surveillance functions (Young and Denovan-Wright, 2021). This phenotypic reprogramming initiated from the transcriptional source severs the excessive phagocytic behavior targeting interneuron synapses at the execution end.

#### 5.2.2 Synergistic reconstruction of synaptic protection networks

**Figure 4:**
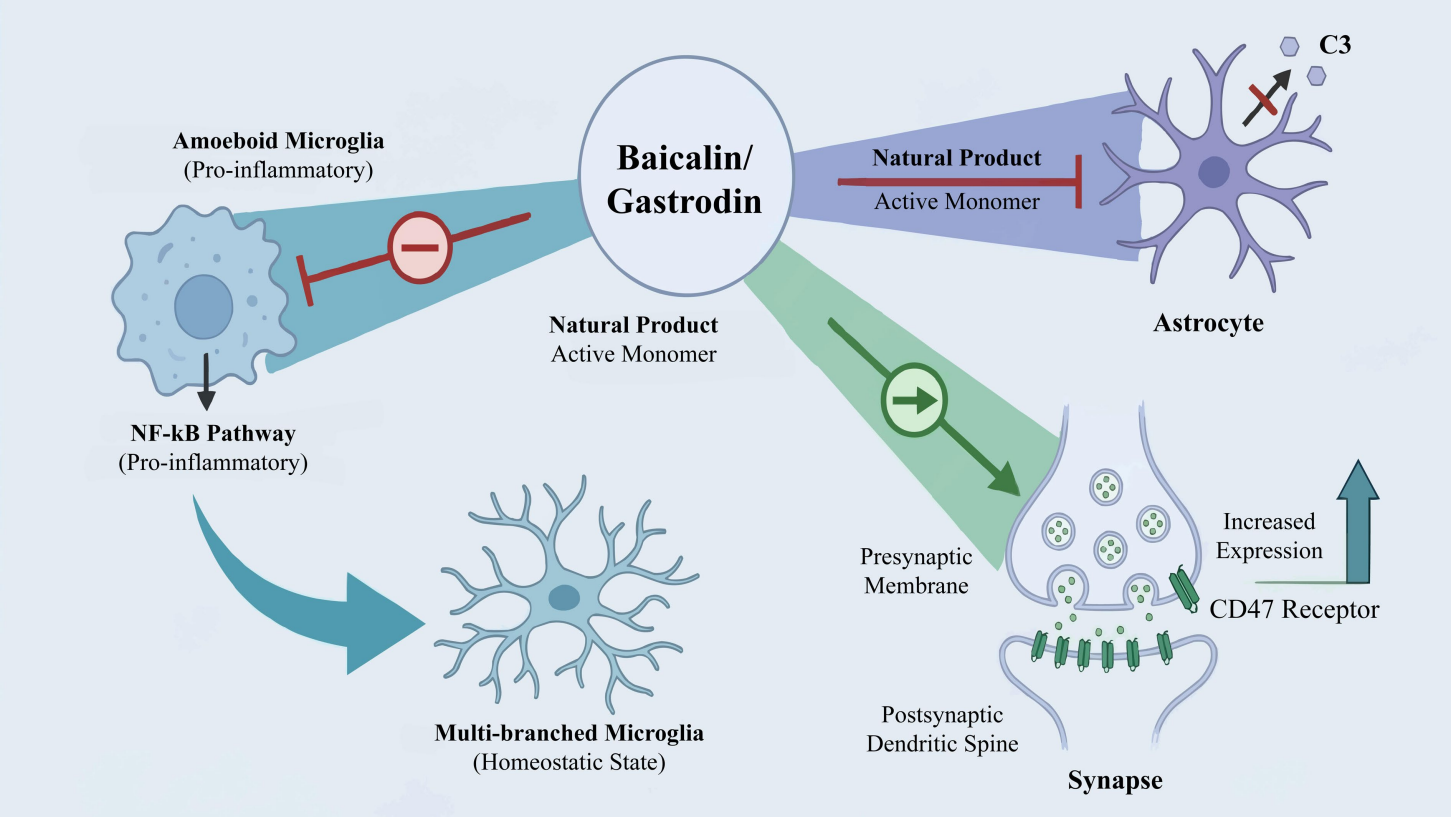
Theoretical multi-target neuroimmune remodeling mediated by natural product active monomers. Natural compounds such as baicalin and gastrodin exert pleiotropic regulatory effects across the multicellular network. Within microglia, these monomers inhibit the pro-inflammatory NF-κB signaling pathway, driving a phenotypic transition from an invasive pro-inflammatory ameboid state to a multi-branched, homeostatic protective state (Yang et al., 2024b). Concurrently, they act on astrocytes to suppress the secretion of complement C3 into the microenvironment. At the synaptic interface, these compounds specifically upregulate the expression of the CD47 receptor on the presynaptic membrane. This coordinated multi-node intervention simultaneously attenuates pathological complement tagging and restores intrinsic synaptic defense signals (Miao et al., 2023).

The intervention of natural compounds is not limited to the single target of microglia but can synergistically reorganize the local protection network of multicellular interactions (Ha et al., 2025). At the synaptic defense end, specific active monomers have been proven to directly increase the expression abundance of CD47 on the presynaptic membrane (Sun et al., 2025). The restoration of CD47 density reactivates the SIRPα inhibitory receptor signaling on the microglial surface, reconstructing the molecular defense barrier of the synapse (Wang et al., 2025). At the microecological fine-tuning end, these compounds can penetrate the inflammatory transcriptional network of astrocytes, dynamically reducing the synthesis and secretion levels of their complement C3. The reduction in astrocyte-derived C3 release directly blocks the pathological C3b deposition on the synaptic surface (Zhang et al., 2025b). This bidirectional synergistic effect of synchronously weakening the “eat me” tag and strengthening the “immune evasion” signal precisely repairs the local neuroimmune homeostasis in the striatum (Figure 4) (Wu et al., 2025a).

### 5.3 Smart nanodelivery platforms hold promise for driving precise intervention processes across the BBB

Natural product monomers face the translational problem of low BBB penetration rates during systemic administration. Smart nanodelivery systems provide an ideal physical carrier to solve this neuropharmacokinetic challenge.

#### 5.3.1 Receptor-mediated transcytosis (RMT) nanotransport strategies across the BBB

Utilizing engineered liposomes and natural exosomes modified with transferrin receptor-binding peptides or specific neurotropic polypeptides can achieve highly efficient targeted delivery across the barrier (Yang et al., 2023). This transport relies heavily on receptor-mediated transcytosis (RMT). In the blood circulation, nanocarriers specifically recognize and lock onto highly expressed receptors on the luminal surface of brain microvascular endothelial cells (Buendia-Corona et al., 2026). Following receptor-ligand binding, the clathrin- or caveolin-mediated endocytosis process is directly initiated (Zhu et al., 2023). Nanovesicles are engulfed into cellular endosomes, directionally cross the endothelial cell cytoplasm along the cytoskeleton, and ultimately undergo membrane fusion and release at the basement membrane side (Arguello et al., 2021). This active transport mechanism extensively elevates the absolute concentration of therapeutic monomers in the striatal lesion area (Sun et al., 2025).

#### 5.3.2 Smart responsive drug release mechanisms targeting activated microglia

**Figure 5:**
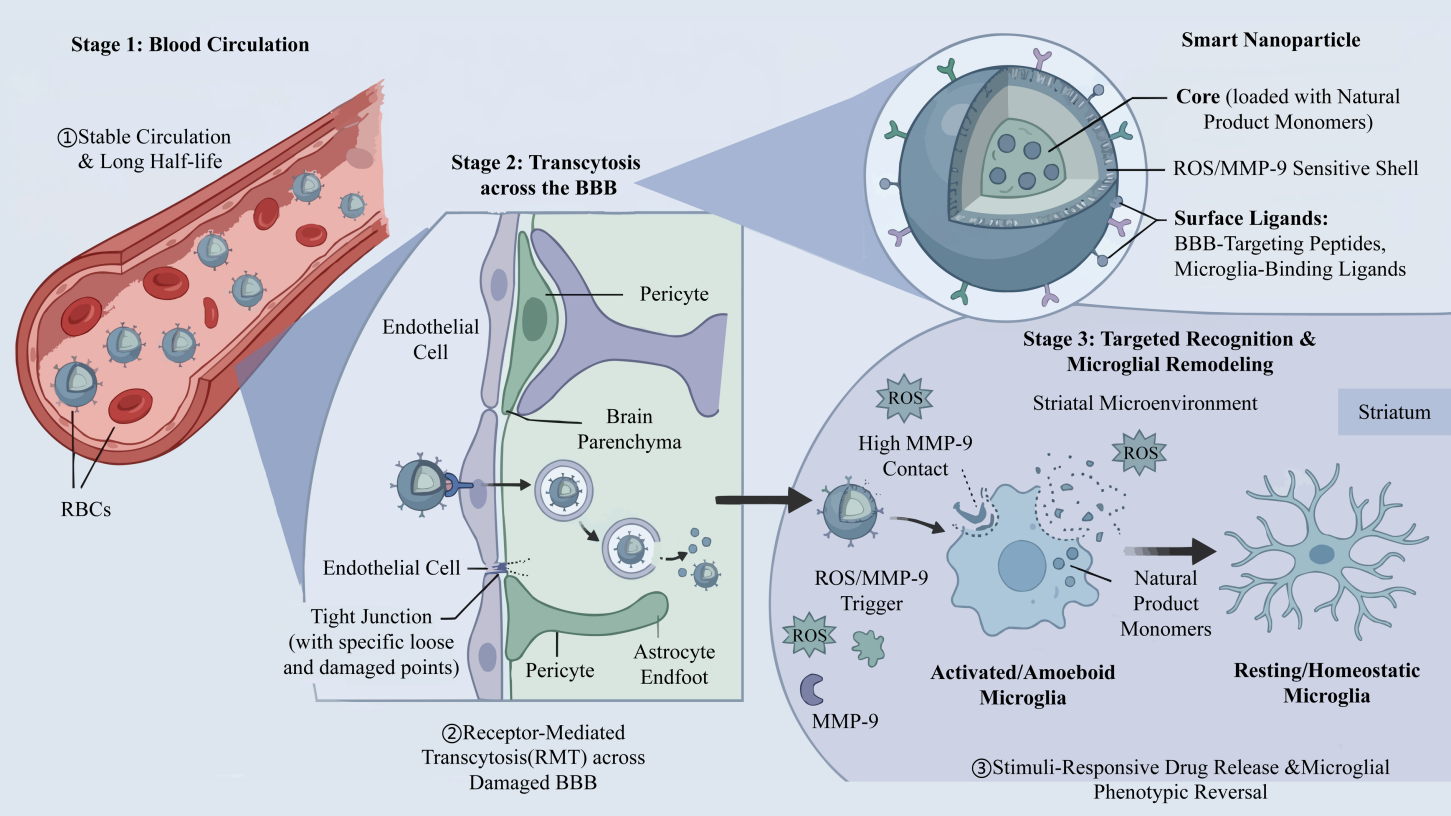
Proposed conceptual model for the spatiotemporal precision delivery of natural monomers via smart nanodelivery platforms in tourette syndrome. Stage 1: blood circulation. Engineered smart nanoparticles, comprising a natural monomer-loaded core and a stimuli-sensitive shell modified with targeting ligands, maintain stable circulation and a long half-life within the systemic vasculature (Song et al., 2024). Stage 2: transcytosis across the BBB. Utilizing surface-modified BBB-targeting peptides, the nanocarriers penetrate the brain parenchyma via transcytosis across the BBB, specifically leveraging regions of increased permeability within the developmental NVU (Wu et al., 2023b; Prades et al., 2025). Stage 3: targeted recognition and microglial remodeling. Upon entering the striatal microenvironment, the pathological accumulation of ROS and MMP-9 triggers the responsive disassembly of the nanoparticle shell (Zhang et al., 2025a). This stimuli-sensitive mechanism induces the localized burst release of natural product monomers in the vicinity of activated microglia (Li et al., 2025a). The therapeutic cargo subsequently promotes microglial phenotypic reversal from a pro-inflammatory ameboid state to a resting homeostatic state, achieving precise intervention with minimized systemic off-target effects (Bellavita et al., 2025). This schematic represents a hypothesized therapeutic trajectory based on the unique neuroinflammatory characteristics of the TS striatum, pending empirical validation in specific *in vivo* models.

To avoid premature drug leakage in non-target areas or the induction of systemic immunosuppression, smart nanoplatforms introduce stimuli-responsive disassembly mechanisms matching the biochemical characteristics of the lesion (Bellavita et al., 2025). The impaired striatal microenvironment in TS is characterized by significant local accumulation of ROS and high expression of MMPs (such as MMP-9) (Murgia et al., 2026). Engineered carrier shells can precisely embed specific chemical bonds, such as ROS-sensitive thioketal bonds or MMP-specific substrate polypeptides (Wang et al., 2023). Once the nanodrugs cross the BBB and enter the target area, the high concentration of pathological metabolites can specifically cleave the aforementioned responsive chemical bonds. The carriers subsequently undergo structural disassembly or phenotypic inversion, instantaneously burst-releasing the internally loaded active monomers (Wu et al., 2023a). This spatiotemporally dual-targeted drug delivery mode precisely restricts drug release to the vicinity of abnormally activated microglia, achieving a precise balance between therapeutic concentration and systemic safety (Figure 5) (Wang et al., 2023). Crucially, by confining the inhibition of microglial phagocytosis strictly to the pathological striatal microenvironment, this responsive mechanism theoretically spares homeostatic microglia in non-target brain regions (such as the cerebral cortex and hippocampus). Consequently, the physiological synaptic pruning essential for normal infant brain development and cognitive maturation remains entirely undisrupted (Faust et al., 2021; Soteros and Sia, 2022), significantly minimizing the risk of adverse behavioral or developmental side effects (Borreca et al., 2024).

#### 5.3.3 Current empirical limitations and essential preclinical trajectories

Despite the theoretical advantages of engineered nanoplatforms, there is currently a lack of empirical data—either from clinical trials or animal models—supporting their specific efficacy in TS. The strategies discussed above must be regarded as a hypothetical framework rather than an imminent translational reality. To bridge this translational gap, several critical preclinical analyses are urgently required.

Initial experimental validations must focus on targeted BBB penetration and biodistribution in TS-specific animal models (such as Hdc knockout or MIA-induced models) to confirm whether nanocarriers can efficiently accumulate in the striatum during early developmental windows (Prades et al., 2025). Furthermore, the *in vivo* trigger-release efficacy requires quantitative assessment. Researchers should employ advanced in vivo molecular imaging to verify whether local ROS or MMP-9 levels in the TS striatum are sufficient to initiate the responsive disassembly of nanocarriers (Zhang et al., 2025a). Beyond pharmacodynamics, rigorous developmental neurotoxicity (DNT) and safety analyses remain paramount (El-Beltagy et al., 2026). Given that TS is a pediatric-onset disorder, the long-term impact of nanomaterials on physiological synaptic pruning, off-target cellular toxicity, and overall brain maturation must be systematically evaluated before any clinical translation can be considered.

### 5.4 Systematic summary and translational challenge assessment of cutting-edge intervention strategies

To systematically evaluate the current pharmacological progress targeting the neuroimmune network and its druggability, it is necessary to comprehensively outline the core characteristics of various intervention methods in their mechanisms, research and development progress, and clinical applications (Sun et al., 2025). From classic broad-spectrum glial modulators to highly cutting-edge nano-assembly systems, translational strategies across different dimensions exhibit distinct stepwise enhancement characteristics in molecular mechanisms and spatial targeting (Amorelli and Martino, 2026). Outlining the similarities and differences of these cutting-edge schemes in indicators such as BBB penetration efficiency, off-target risk, and developmental safety holds promise for providing a clear direction to identify the most promising next-generation TS intervention drugs (Woerner et al., 2025). The following table summarizes potential intervention strategies based on the current research consensus (Table 2).

**Table 2:** Comparison of potential frontier interventional strategies targeting neuroimmune dysregulation within the CSTC circuitry.

Intervention strategy category	Representative drugs/agents	Core targets and regulatory mechanisms	Primary evidence origin	Current research stage	Proposed advantages and limitations in TS intervention
Classical glial modulators	Minocycline	Inhibits the p38 MAPK pathway to attenuate pro-inflammatory factor release	General neuroinflammation and clinical trials in other neurological disorders (Tansey et al., 2022; Mohammady et al., 2026)	Phase II/III clinical trials	Possesses broad accessibility but lacks spatial specificity; long-term developmental safety remains uncertain
Specific complement blockers	CR3 antagonists	Obstructs microglial receptor recognition and phagocytosis of C3-tagged synapses	General neurodegenerative and depression/stress models (Han et al., 2024; Cai et al., 2026b)	Validation in rodent models	Theoretically offers targeted mechanisms, but prone to interfering with physiological pruning in developing brains
Multi-target natural products	Baicalin, gastrodin, etc.	Synergistically inhibits NF-κB-mediated phenotypic activation, inhibits C3 secretion, and upregulates CD47	Broad neuroinflammatory models (Yang et al., 2024b; Cai et al., 2026a), alongside limited TS-specific preclinical models (Sun et al., 2025)	In vitro and preclinical models	Conceptually capable of multi-node microenvironmental remodeling, but specific efficacy in TS models requires validation.
Engineered nanodelivery systems	Targeting peptide-modified exosomes/liposomes	Achieves precise drug delivery across the BBB targeting pathological glial cells	Advanced drug delivery and oncology fields (Wu et al., 2023b; Yang et al., 2023; Bellavita et al., 2025)	Proof-of-concept / prospective conceptualization	Hypothesized to significantly reduce systemic adverse effects, but strictly conceptual pending TS-specific in vivo testing

Adapted from Gao et al. (2024).

## 6 Future perspectives

### 6.1 Summary of core hypothesis and theoretical value of neuroimmune microenvironment remodeling

In summary, the proposal of the microglia-mediated abnormal synaptic pruning hypothesis has significantly advanced our understanding of the pathogenesis of TS, successfully positioning it as a key biological hub connecting genetic susceptibility, developmental immune stress, and neural circuit structural defects. This review not only systematically outlines the molecular machinery from immune priming to synaptic destruction; more importantly, we established and deeply explored a novel conceptual framework of dynamic, complex, and bidirectional interactions between microglia and the CSTC circuit. We demonstrated in detail how abnormal synaptic pruning may act as a core pathological driving force, extensively reconstructing the overall electrophysiological landscape of the basal ganglia by physically eliminating inhibitory interneurons within the striatum. Concurrently, we elucidated how the inherent neuronal metabolic characteristics and unique matrix structure of the striatum actively and finely set the sensitivity threshold of specific synapses to immune attacks through complex biochemical signals. Comprehensively and extensively understanding this intricate, mutually causal dynamic relationship is by no means merely a theoretical exploration; it constitutes a crucial theoretical cornerstone for us to truly rationally design next-generation intervention therapies with disease-modifying potential.

### 6.2 Key scientific questions in neurodevelopmental immunology urgently requiring resolution

Although current immunological research on neurodevelopmental disorders has made significant progress, the field of TS remains in its infancy regarding the elucidation of molecular details and still faces a series of major scientific questions in clinical translation. First, future research must transcend simplified two-dimensional cell cultures or static brain slice models, utilizing human induced pluripotent stem cells (hiPSCs)-derived brain organoids or high-fidelity animal models to definitively confirm the exact contribution of microglia in the natural developmental process of the striatum and their true responses to pathological triggers. Specifically, future studies must prioritize obtaining direct empirical evidence within TS-relevant models to verify: (1) *in vivo* microglial engulfment of PV + FSI synapses; (2) targeted complement deposition on these specific terminals; (3) the pathological downregulation of protective signals like CD47; and (4) the definitive causal relationship between aberrant pruning and macroscopic tic-like phenotypes. Second, comprehensively and clearly mapping the differential sensitivity atlas of different neuronal subtypes within the striatum to complement tagging and microglial phagocytosis is a necessary prerequisite for achieving precision targeted therapy in the future. Furthermore, achieving spatiotemporal specificity remains a critical translational challenge. Future investigations must evaluate how to selectively suppress aberrant microglial phagocytosis in the basal ganglia without inadvertently impairing physiological synaptic pruning in the developing infant brain. Non-specific inhibition of microglial activity during early childhood carries the known risk of inducing secondary cognitive or behavioral deficits (Matuleviciute et al., 2023). Therefore, the long-term impact of targeted anti-pruning interventions on overall brain maturation must be systematically monitored (Paolicelli et al., 2022). Third, the immune remodeling potential based on natural products and nanomedicine currently remains highly dependent on logical deduction and preliminary experiments; their actual pharmacodynamic intensity after crossing the BBB and their impact on long-term nervous system homeostasis still require large-scale, long-term in vivo experiments for definitive verification. Finally, from the perspective of drug development, there is currently an extreme lack of pharmacological modulators capable of specifically regulating complement pathway components with minimal systemic toxicity; developing such next-generation drugs is a critical prerequisite for achieving safe and effective clinical applications.

### 6.3 Core limitations of the proposed conceptual framework

While the microglia-mediated synaptic pruning hypothesis offers a compelling structural perspective for TS, it is imperative to explicitly acknowledge the fundamental limitations of the proposed model. First, there remains a profound lack of direct human post-mortem evidence confirming microglial engulfment of specific interneuron synapses within the TS basal ganglia; the molecular execution sequence described herein relies heavily on extrapolations from other neurodegenerative and stress models. Second, TS is characterized by profound genetic and clinical heterogeneity. Utilizing rare HDC mutations as a primary mechanistic prototype may not fully capture the diverse pathogenesis of the broader polygenic patient population. Third, current *in vivo* TS models (such as Hdc knockout or MIA-induced mice) exhibit inherent biological limitations and cannot completely replicate the complex spatiotemporal dynamics of the human CSTC circuitry. Fourth, the role of environmental triggers, particularly immune-triggered tic exacerbation and PANDAS/PANS-related mechanisms, remains highly controversial, with ongoing uncertainty regarding whether these infections represent direct causal triggers or merely non-specific stress modifiers.

Therapeutically, the proposed translational interventions face equally severe hurdles. A paramount safety concern is the profound risk of interfering with physiological synaptic pruning during early childhood development, which could inadvertently induce secondary cognitive or behavioral delays. Furthermore, achieving the spatiotemporal precision required to selectively target pathological microglia in vivo, without triggering broad systemic immunosuppression or disrupting homeostatic glial functions, remains a significant technological challenge. Finally, while natural products and nanomedicine offer theoretical multi-target advantages, their clinical translation is currently limited by an extreme lack of rigorous, TS-specific pharmacokinetic data, unknown long-term developmental neurotoxicity profiles, and the inherent complexities of engineered nanomaterial scale-up. Addressing these multi-layered limitations must be the absolute priority for future empirical investigations.

### 6.4 Future research directions and clinical translation perspectives from a multidisciplinary viewpoint

Overcoming the aforementioned severe challenges and driving the advancement of the TS treatment paradigm will rely heavily on the comprehensive application of multidisciplinary approaches and the specific application of a series of cutting-edge technologies. Utilizing cutting-edge technologies such as single-cell multi-omics and spatial transcriptomics will assist us in systematically resolving intercellular differences in immune priming at an unprecedented resolution within the intact neural microenvironment, and in accurately delineating the spatiotemporal atlas of the dynamic evolution of synaptic pruning. By utilizing advanced multiphoton in vivo imaging technology, we hold the promise to monitor the microscopic interaction processes between microglia and FSIs in real time, thereby capturing the earliest signals of disease onset.

Applying nanomedicine to develop smart delivery systems can directionally deliver active monomers from natural products or precise immunomodulatory molecules to the striatum to achieve the translational goal of enhancing efficacy and reducing toxicity. More importantly, the significance of the fundamental biological principles revealed by this hypothesis may extend far beyond TS itself. Future research should actively explore the universal applicability of this paradigm—where metabolic susceptibility induces abnormal pruning—in other diseases involving neuroimmune homeostatic imbalance, such as OCD, ASD, or early-onset neurodegenerative diseases. By systematically and persistently overcoming these key problems in basic and translational medicine, we have reason to believe that the scientific community will fully uncover the immense therapeutic potential underlying the strategy of targeting the neuroimmune microenvironment, and ultimately translate it into innovative therapies that bring tangible clinical benefits to the vast number of patients with tic disorders.

## 7 Conclusion

In summary, microglia-mediated abnormal synaptic pruning not only provides a novel pathological perspective for exploring the pathogenesis of TS but also logically bridges the gap between genetic susceptibility and clinical neurotransmitter dysregulation. As a key node in the neuroimmune interaction network, the phenotypic transformation of microglia from physiological synaptic surveillance to pathological excessive pruning is hypothesized to be a contributing factor in the structural impairment of inhibitory interneurons within the CSTC circuit. This hypothesis model based on microenvironmental remodeling provides a framework to view TS as a circuit microstructural developmental disorder rather than solely a neurotransmitter imbalance.

Looking forward to future clinical translation pathways, theoretically, we should primarily rely on high-resolution in vivo molecular imaging technologies, such as upgraded TSPO-PET imaging targeting microglial activation, to establish a dynamic correlation between disease progression and immune microenvironmental evolution at the in vivo level. Concurrently, leveraging human 3D brain organoid models, we hold the promise to verify the efficacy of innovative drugs targeting the complement cascade or defense signaling axes within microsystems that more closely approximate the human genomic background. Through the profound integration of multidisciplinary approaches and the intervention of precision medicine, therapeutic strategies targeting the neuroimmune network hold the promise to break through existing pharmacological bottlenecks, opening a highly promising clinical pathway for achieving early warning, precise diagnosis, and disease-modifying therapy for TS.
